# Overexpression of hnRNPC2 induces multinucleation by repression of Aurora B in hepatocellular carcinoma cells

**DOI:** 10.3892/ol.2013.1167

**Published:** 2013-01-31

**Authors:** DA-QUAN SUN, YING WANG, DING-GAN LIU

**Affiliations:** State Key Laboratory of Molecular Biology, Institute of Biochemistry and Cell Biology, Shanghai Institute for Biological Sciences, Chinese Academy of Sciences, Shanghai 200031, P.R. China

**Keywords:** heterogeneous ribonuclear protein C2, multinucleation, hepatocellular carcinoma cell, Aurora B, eukaryotic translational initiation factor 4E

## Abstract

Heterogeneous ribonuclear protein C2 (hnRNPC2), an RNA binding protein, is a component of hnRNPC which is upregulated in many tumors. Multinucleation exists in many tumors and is positively correlated with tumor grade. To uncover the correlation between hnRNPC2 and multi-nucleation in hepatocellular carcinoma SMMC-7721 cells, we constructed a pEGFP-hnRNPC2 vector and transfected it into cancer cells. Our results revealed that overexpression of hnRNPC2 induced multinucleation in SMMC-7721 cells. Tracking tests indicated that the induced multinucleated cells were unable to recover to mononuclear cells and finally died as a result of defects in cell division. Furthermore, Aurora B, which was localized at the midbody and plays a role in cytokinesis, was repressed in hnRNPC2-overexpressing cells, whose knockdown by RNA interference also induced multinucleation in SMMC-7721 cells. Quantitative polymerase chain reaction (qPCR) and mRNA-protein co-immunoprecipitation results revealed that Aurora B mRNA did not decrease in hnRNPC2-overexpressing cells, instead it bound more hnRNPC2 and less eIF4E, an mRNA cap binding protein and translational initiation factor. Moreover, hnRNPC2 bound more eIF4E in hnRNPC2-overexpressing cells. These results indicate that hnRNPC2 repressed Aurora B binding with eIF4F, which must bind with Aurora B mRNA in order to initiate its translation. This induced multinucleation in hepatocellular carcinoma cells. In addition, hnRNPC2 accelerated hepatocellular carcinoma cell proliferation. Collectively, these data suggest that hnRNPC2 may be a potential target for hepatocellular carcinoma cell diagnosis and treatment.

## Introduction

Heterogeneous ribonuclear protein C (hnRNPC) is an RNA-binding protein located in the nuclei of normal cells; however, it is also distributed in the cytoplasm of tumor cells (1). It is thought to be a prognostic marker in tumors (2,3). hnRNPC has two isoforms, C2 and C1, coded by a single gene and generated by alternative splicing of the same transcript. The difference between the two isoforms is that C2 has an additional 13 amino acid insert after Ser^107^(4). hnRNPC plays multiple roles in post-transcriptional regulation, including alternative splicing (5), nuclear retention and export (6), stability (7,8) and translation (3,9,10). Several studies have shown that hnRNPC is overexpressed in tumors, including hepatocellular carcinoma and breast cancer (2,11). When its expression is repressed, tumor growth is suppressed and occasionally inhibited (12,13).

Another important characteristic of tumors is pleomorphism, including multinucleation, particularly in high grade tumors (14,15). In humans, the vast majority of normal cells are mononuclear except a few specific types of cells, including hepatocytes (16). Although multinucleation is a normal phenomenon in adult liver with age, pathogens, including virus infection and carcinogens, are indispensible elements to accelerate this process (17–19). Multinucleation is the result of a change or disorder in gene regulation whether for normal cell development progression or for disease (16,20,21). Among these genes, Aurora B is essential to chromosome segregation and cytokinesis. It is an important component of the chromosomal passenger complex and plays multiple roles in cell division such as mitotic spindle assembly, kinetochore assembly, regulation of mitotic checkpoints, chromosome compaction in anaphase and regulation of cleavage furrow ingression (20–22). During these processes, Aurora B is located at the midbody in late anaphase and cytokinesis to recruit substrates that are necessary for cytokinesis and exerts enzymatic activity to complete cytokinesis (23–26). Upregulation of Aurora B and its repression lead to cytokinesis failure and induced multinucleation (27–29).

In this study, we found that hnRNPC2 is correlated with multinucleation in hepatocellular carcinoma SMMC-7721 cells. Further investigation revealed that hnRNPC2 induced multinucleation by repressing the expression of Aurora B.

## Materials and methods

### Materials

The eukaryotic translational initiating factor 4E (eIF4E) antibody and protein A/G-agarose were purchased from Bioworld (Uitgeest, The Netherlands). The Aurora B antibody and hnRNPC2 antibody were purchased from Epitomics (Burlingame, CA, USA). TRIzol, Lipofectamine 2000 and RPMI-1640 were purchased from Invitrogen Life Technologies (Carlsbad, CA, USA). The PrimeScript™ reverse transcription-polymerase chain reaction (RT-PCR) kit was purchased from Takara Bio, Inc. (Shiga, Japan). Taq Platinum DNA polymerase was purchased from Tiangen (Beijing, China). pEGFP-C1 was purchased from Clontech Laboratories (Mountain View, CA, USA). Primer synthesis and DNA sequencing were performed by SunnyBio. (Shanghai, China). siRNA was supplied by Genepharma (Shanghai, China). Propidium iodide (PI) was purchased from Beyotime (Jiangsu,China). 4,6-diamino-2-phenyl indole (DAPI) was purchased from Sigma (St. Louis, MO, USA). The cell counting kit (CCK)-8 was purchased from Dojindo (Kumamoto, Japan). iQ™ SYBR®-Green supermix was purchased from Bio-Rad (Hercules, CA, USA). SMMC-7721 cells, HL-7702 cells, A549 cells and BT549 cells were from the cell bank of the Chinese Academy of Sciences.

The study was approved by the Ethics Committee of the Institute of Biochemistry and Cell Biology, Shanghai Institutes for Biological Sciences, Chinese Academy of Sciences, Shanghai, China.

### RNA extraction, cDNA synthesis and expressional vector construction

SMMC-7721 cells (60 mm dish) were lysed by 1 ml TRIzol following 3 washes with phosphate-buffered saline (PBS) to extract the total RNA, following the manufacturer’s instructions. cDNA synthesis was performed using the PrimeScript RT-PCR kit, according to the manufacturer’s instructions and DNA amplification was performed by Taq Platinum DNA polymerase with primers as followed: hnRNPC (NM_001077442), 5′-ACCTCGAGACACGATGGCCAGCAACGTT-3′, 5′-CAG AATTCGCTTAAGAGTCATCCTCGCC-3′. The amplified hnRNPC cDNA fragment was T-A cloned into a pMD18-T vector. DNA sequencing was used to obtain the hnRNPC2 gene, which was inserted into the pEGFP-C1 vector between the restriction sites *Xho*I and *Eco*RI.

### Cell culture, DNA transfection and cell screening

Cells were cultured in RPMI-1640 medium plus 10% newborn bovine serum (full medium). Cells were seeded in a 24-well plate (1.5×10^5^ cells per well) 24 h before transfection. For transfection, 1.0 *μ*g plasmid was used per well. Transfection was performed according to the manufacturer’s instructions. The transfectants were screened by full medium plus 800 *μ*g/ml geneticin for 7 days and cultured in full medium plus 400 *μ*g/ml geneticin for a further 14 days. All cell colonies displaying green fluorescence were obtained under a fluorescent microscope and cultured together for proliferation.

### Cell counting

Cell counting was performed according to the instructions of the CCK-8 kit in a 96-well plate.

### RNA interference

RNA interference was performed according the instructions of Lipofectamine 2000. For each 35 mm dish 600 pmol siRNA was used. The siRNA-Aurora B sequence was according to the literature (30). At 72 h post-transfection, cells were detected by flow cytometry and western blotting.

### Western blotting

Western blotting was performed according to the literature (31).

### PI staining and flow cytometry

Following 3 washes with PBS (pH 7.2), cells cultured on cover slips or digested by 0.25% trypsin were fixed with ice-cold 1.25% paraformaldehyde for 30 min [this step is only for green fluorescent protein (GFP) or GFP fusion protein expressed cells and their control cells]. Then, the fixed cells were washed twice with PBS and fixed with ice-cold 75% ethanol for 2 h on ice. Prior to staining with 5 *μ*g/ml PI, cells were digested by 30 *μ*g/ml RNase A at 37°C for 30 min. Finally, cells were observed under a fluorescence microscope or detected by flow cytometry.

### Immunofluorescence staining and laser scanning confocal microscopy

Cells were seeded on cover slips in a 24-well plate 24 h before transfection. At 72 h post-transfection, cover slips with cells adhered to the surface were washed with PBS and fixed with 4% paraformaldehyde for 40 min at room temperature. Then, cells were permeabilized with 1% Triton X-100 for 5–10 min at room temperature and blocked by 5% skimmed milk for 1 h at 37°C. Next, cover slips were incubated with the primary antibody for 12 h then the secondary antibody labeled with rhodamine for 8 h at 4°C. After staining with 1 *μ*g/ml DAPI in methanol for 4 min at room temperature, cover slips were sealed with antifade mounting medium. These stained cells were observed under a Leica TCS-SP laser scanning confocal microscope.

### mRNA-protein co-immunoprecipitation and protein-protein co-immunoprecipitation

mRNA-protein co-immunoprecipitation (co-IP) was performed according to the protocol (32). Protein-protein co-IP was performed according to the above protocol with certain modifications: 6 *μ*g/ml RNases and 4 U/ml DNase were substituted for the RNase inhibitor and the extract was incubated at 37°C for 30 min to digest DNA and RNA. Following co-IP, the harvested protein A/G agarose was mixed with sodium dodecyl sulphate (SDS) loading buffer and incubated at 50°C for 30 min. It was then centrifuged at 4000 rpm for 5 min and the supernate was used for immunoblot analysis.

### Real-time quantitative PCR

cDNAs were synthesized as mentioned above. The real-time PCR reaction procedure was performed as follows: 95°C for 2 min; cycle: 95°C for 20 sec, 55°C for 30 sec and 72°C for 30 sec; annealing from 65°C to 95°C with 0.5°C progressive increases. The primers used in this study were: Aurora B (NM_004217): 5′-ATAGCAGTGGGACACCCGACAT-3′ and 5′-GGGACTTGAAGAGGACCTTGAGC-3′; p190-B (NM_001030055): 5′-ATTTGACCTCCTGAGCACTT-3′ and 5′-TGTAGGCTTCATCCTCCATA-3′; glyceraldehyde 3-phosphate dehydrogenase (GAPDH, NM_002046): 5′-CCTGTTCGACAGTCAGCCGCATC-3′ and 5′-CGACCA AATCCGTTGACTCCGACC-3′.

### Statistical analysis

Each experiment was repeated at least three times and data were analyzed by analysis of variance test. P<0.01 was considered to indicate a statistically significant difference.

## Results

### Overexpression of hnRNPC2 induced multinucleation in human hepatocellular carcinoma cells

**Figure 1:**
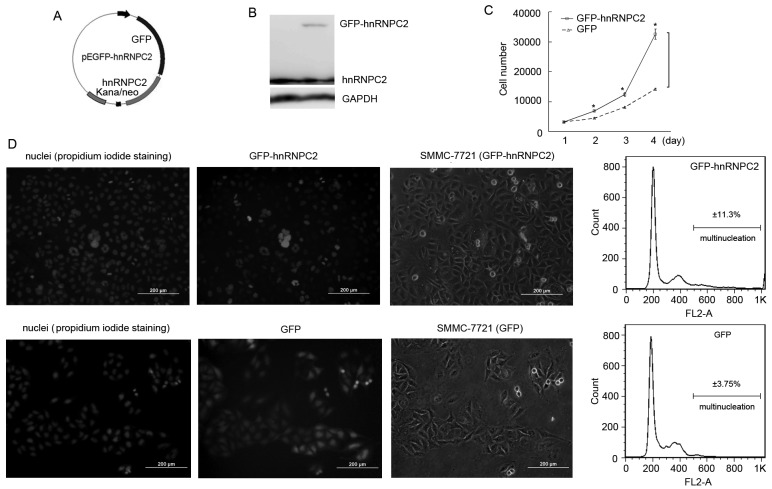
hnRNPC2 induces multinucleation in hepatocellular carcinoma SMMC-7721 cells. (A) Eukaryotic expressional vector pEGFP-hnRNPC2. The hnRNPC2 cDNA was inserted into the restriction sites between *Xho*I and *Eco*RI. (B) Exogenous fusion protein GFP-hnRNPC2 was expressed in transfected SMMC-7721 cells, detected by western blotting. (C) The proliferative curve of cells. GFP represented GFP expressed SMMC-7721 cells; GFP-hnRNPC2 represented GFP-hnRNPC2 expressed SMMC-7721 cells. (D) Overexpression of hnRNPC2 induced multinucleation in SMMC-7721 cells detected by fluorescent microscopy and flow cytometry. ^*^P<0.01. GFP, green fluorescent protein; hmRNPC, heterogeneous ribonuclear protein C; GAPDH, glyceraldehyde 3-phosphate dehydrogenase.

To reveal whether there was a positive correlation between hnRNPC2 and multinucleation, we constructed a eukaryotic expressional vector pEGFP-hnRNPC2 (Fig. 1A) and transfected it into hepatocellular carcinoma SMMC-7721 cells. Western blot results revealed that GFP-hnRNPC2 is expressed at 48 h post-transfection (Fig. 1B). Following screening by geneticin, GFP-hnRNPC2-expressed cell colonies were all obtained under fluorescent microscope and mixed into one culture. Then, cell proliferative rate tests were carried out and the results revealed that the exogenous hnRNPC2-expressed cells (hnRNPC2 overexpression) accelerated their proliferation (Fig. 1C). Notably, under the fluorescent microscope, we found that a number of those cells showed more than two nuclei, glowing green fluorescence, in a cell with an expanded cytoplasm (Fig. 1D). To evaluate the number of cells with multinucleation as a result of overexpressed hnRNPC2, fluorescent microscopy and flow cytometry were used. As Fig. 1D shows, hnRNPC2-overexpressing SMMC-7721 cells possessed more multinucleated cells; nearly 11.3% cells were induced to multinucleation, while the control cells showed 3.8% multinucleated cells. Furthermore, when pEGFP-hnRNPC2 was transfected into breast cancer BT549 cells and noncancerous hepatocellular HL-7702 cells, they showed similar results (data not shown). Collectively, these results indicate that overexpression of hnRNPC2 is capable of inducing multinucleation in hepatocellular carcinoma cells and this effect may be universal.

### Destiny of multinucleated cells induced by overexpressed hnRNPC2

**Figure 2:**
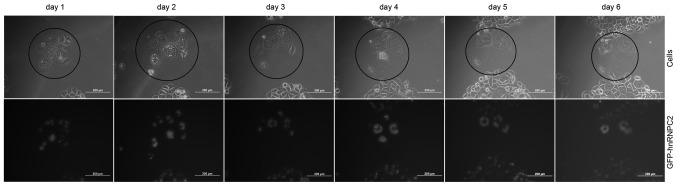
Multinucleated cells induced by GFP-hnRNPC2 died due to a lack of cytokinesis. The induced multinucleated cells (in black circles) were continuously observed under a fluorescent microscope to track their progression. They lost the ability to divide and instead increased in nuclear number and underwent cytoplasm expansion, resulting in cell death. This was a typical group of figures in 8 repeat tracking tests. hnRNPC2, heterogeneous ribonuclear protein C2.

We demonstrated that multinucleated cells were induced by the overexpression of hnRNPC2. To elucidate the destiny of the induced multinucleated cells, we tracked the process of the induced multinucleated cells’ progression every 24 h using a fluorescent microscope. As Fig. 2 shows, the induced multinucleated cells lose the ability to divide and they do not recover back to mononuclear cells. Instead, they increase in nuclear number and undergo maximal expansion of their cytoplasm. As time lapsed, they became giant multi-nucleated cells and finally, due to an inability to divide, they died. In 8 groups of tracking tests, all multinucleated cells died. We conclude that the induced multinucleated cells lose the ability to divide and therefore die.

### Aurora B was repressed in hnRNPC2-overexpressing cells

**Figure 3:**
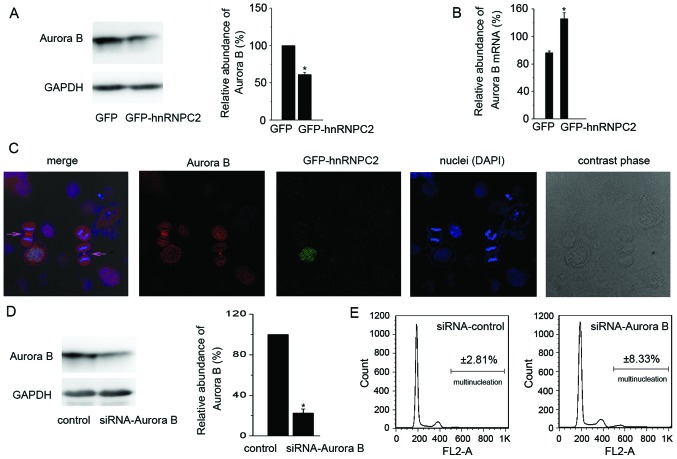
The Aurora B protein was repressed in hnRNPC2-overexpressing cells and it plays a role in cytokinesis. (A) The expression of Aurora B protein was repressed in hnRNPC2-overexpressing SMMC-7721 cells. (B) Aurora B mRNA increased in hnRNPC2-overexpressing SMMC-7721 cells. (C) Aurora B was located in the midbody during cytokinesis. Red, Aurora B stained by rhodamine labeled monoclonal antibody; green, GFP-hnRNPC2; blue, nuclei stained by DAPI. They were detected by laser scanning confocal microscope. (D) RNA interference of Aurora B showed the expression of Aurora B protein was repressed. (E) Deletion of endogenous Aurora B protein by RNA interference induced multinucleation in SMMC-7721 cells. ^*^P<0.01. GAPDH, glyceraldehyde 3-phosphate dehydrogenase; GFP, green fluorescent protein; hnRNPC, heterogeneous ribonuclear protein C; DAPI, 4,6-diamino-2-phenyl indole.

During this study, we found that the expression of the Aurora B protein was repressed in hnRNPC2-overexpressing cells, when compared to the control cells (Fig. 3A). Aurora B localizes at the midzone in late anaphase and recruits and phosphorylates substrates that are essential to complete cytokinesis (23–26). To uncover the importance of Aurora B in cytokinesis of SMMC-7721 cells, immunofluorescent staining and confocal microscopy were carried out together. As Fig. 3C shows, Aurora B is located at the midbody during cytokinesis in SMMC-7721 cells, while GFP-hnRNPC2-expressed multi-nucleated cells lost the ability of cell division. In fact, we did not find an induced multinucleated cell that was in cytokinesis in a series of repeated experiments. Furthermore, we abolished the endogenous Aurora B protein using RNA interference (Fig. 3D). As a result, the percentage of multinucleation in Aurora B knockdown-SMMC-7721 cells increased to 8.3%, compared with 2.8% in the control cells (Fig. 3E). These results indicate that Aurora B plays a vital role in cytokinesis and that overexpression of hnRNPC2 induces multinucleation by repressing the Aurora B protein.

### hnRNPC2 repressed mRNA translation of Aurora B by inhibiting eIF4E binding to its mRNA

**Figure 4:**
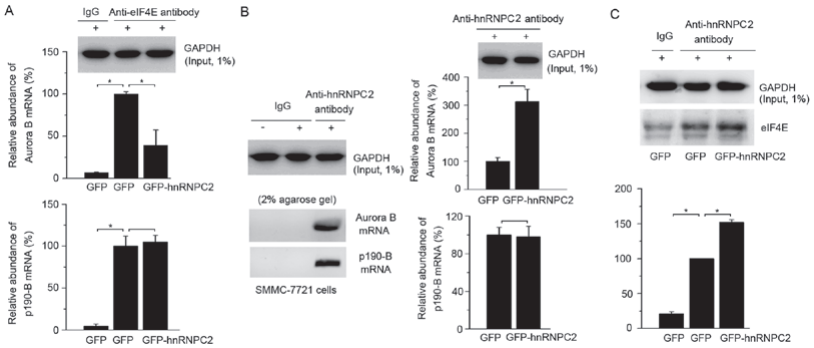
hnRNPC2 repressed Aurora B protein by inhibition of translational initiation. (A) Repressed eIF4E (cap binding protein ) bound with Aurora B mRNA in hnRNPC2-overexpressing cells, while the binding between eIF4E and p190-B mRNA did not change. (B) hnRNPC2 bound specifically with Aurora B mRNA and p190-B mRNA in SMMC-7721 cells and bound more Aurora B mRNA in hnRNPC2-overexpressing cells while little changed in the binding of hnRNPC2 and p190-B mRNA. (C) hnRNPC2 bound more eIF4E in hnRNPC2-overexpressing cells without mRNA and DNA participation. ^*^P<0.01. IgG, immunoglobulin G; eIF4E, eukaryotic translational initiating factor 4E; GFP, green fluorescent protein; hnRNPC, heterogeneous ribonuclear protein C; GAPDH, glyceraldehyde 3-phosphate dehydrogenase.

We demonstrated that overexpression of hnRNPC2 induced multinucleation by repression of the Aurora B protein. Next, we attempted to clarify how hnRNPC2 repressed the expression of Aurora B. First, we examined the Aurora B mRNA in cells. The abundance of Aurora B mRNA increased in hnRNPC2-overexpressing cells, which indicates that the repression of the Aurora B protein is not caused by mRNA transcription but may instead be caused at the translational level (Fig. 3B). To obtain evidence for this hypothesis, we used mRNA-protein co-IP method to detect whether mRNA translation was inhibited in hnRNPC2-overexpressing cells. It is well-known that eIF4E is an mRNA cap binding protein that is necessary for the initiation of cap-dependent mRNA translation (3,33). The mRNA-eIF4E co-IP results revealed that the Aurora B mRNA bound less eIF4E while p190-B mRNA, as a control, did not change in hnRNPC2-overexpressing cells, suggesting that the expression of the Aurora B protein was specifically repressed by translational initiation (Fig. 4A). Furthermore, mRNA-hnRNPC2 co-IP results revealed that Aurora B mRNA specifically bound with hnRNPC2 in SMMC-7721 cells and bound more in hnRNPC2-overexpressing cells, while the relative abundance of hnRNPC2 bound to p190-B mRNA changed little (Fig. 4B). To clarify how hnRNPC2 inhibited eIF4E binding to Aurora B mRNA, protein-protein co-IP was carried out. As expected, hnRNPC2 bound more eIF4E in hnRNPC2-overexpressing cells (Fig. 4C). These results suggest that hnRNPC2 inhibits the binding of eIF4E with Aurora B mRNA by binding with eIF4E, which represses mRNA translational initiation and therefore results in the decrease of the Aurora B protein.

### Roles of hnRNPC2 in hepatocellular carcinoma cells

**Figure 5:**
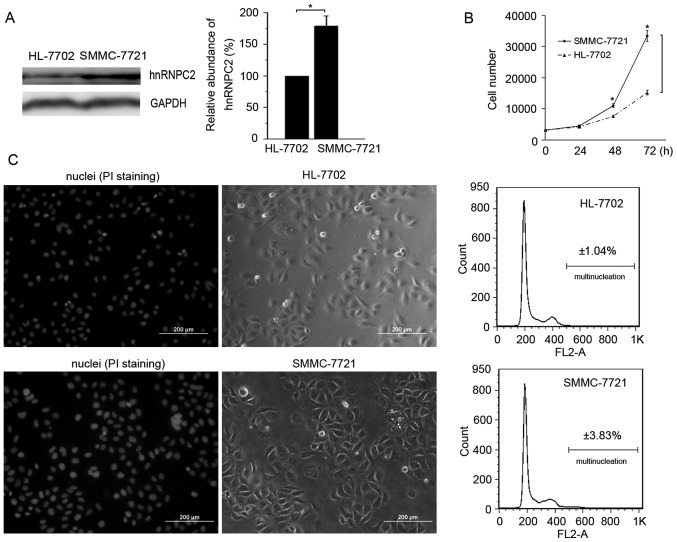
Roles of hnRNPC2 in hepatocellular carcinoma cells. (A) The expression of hnRNPC2 increased in hepatocellular carcinoma SMMC-7721 cells, compared with noncancerous hepatocellular HL-7702 cells. (B) SMMC-7721 cells grew faster than HL-7702 cells. (C) SMMC-7721 cells possessed more multinucleated cells than HL-7702 cells, which were stained by propidium iodide (PI) and detected by fluorescent microscope and flow cytometry. ^*^P<0.01. hnRNPC, heterogeneous ribonuclear protein C; GAPDH, glyceraldehyde 3-phosphate dehydrogenase.

To explore the role of hnRNPC2 in the process of hepatocellular carcinoma cell progression, we examined the expression of hnRNPC2 in noncancerous hepatocellular HL-7702 cells and hepatocellular carcinoma SMMC-7721 cells. Western blotting results revealed that SMMC-7721 cells expressed more hnRNPC2 than HL-7702 cells (Fig. 5A). Cell proliferative rate tests demonstrated that the proliferation of SMMC-7721 cells was much quicker than that of HL-7702 cells (Fig. 5B). Moreover, fluorescent microscopy and flow cytometry results revealed that the percentage of multinucleated cells is three times larger in SMMC-7721 cells when compared with that in HL-7702 cells (Fig. 5C). SMMC-7721 is a low-grade malignant human hepatocellular cell line and hnRNPC and multinucleation are related to tumor grade (2,11,34,35). As mentioned above, we overexpressed hnRNPC2 in SMMC-7721 cells to raise its tumor grade, resulting in an increase in multinucleation (Fig. 1D). These results indicate that the amount of multinucleation increased with increased hnRNPC2, from noncancerous hepatocellular HL-7702 cells to low-grade malignant hepatocellular SMMC-7721 cells and then to SMMC-7721 cells expressing exogenous hnRNPC2, suggesting that hnRNPC2 plays a role in hepatocellular carcinoma cell progression.

## Discussion

Multinucleation is an important characteristic in tumor progression and correlates to tumor grade (35,36). We found that overexpression of hnRNPC2 induced multinucleation in hepatocellular carcinoma cells. From HL-7702 cells to SMMC-7721 cells and then to exogenous hnRNPC2 expressed SMMC-7721 cells, the ratio of multinucleation increased positively with gradually increased expression of hnRNPC2. In this process, Aurora B played an important role to inhibit cytokinesis (23–26,37–40). As indicated, deregulation of Aurora B, either downregulation or upregulation, leads to cytokinesis failure in tumors (27–29,41). Here, we observed that overexpression of hnRNPC2 in SMMC-7721 cells repressed the expression of Aurora B, resulting in cytokinesis failure and multinucleation appearance. This phenomena was also induced by abolishing endogenous Aurora B protein by RNA interference in SMMC-7721 cells, consistent with a previous report (27). Thus, we conclude that hnRNPC2 induces multinucleation by repressing the expression of the Aurora B protein in hepatocellular carcinoma cells. In addition, we found that multinucleation was an irreversible process. Once mononuclear cells were transformed into multinucleated cells, they lost the ability to divide. Instead, they accumulated more nuclei and expanded their cytoplasm and thus became multi-nucleated giant cells and eventually died. As elucidated above, Aurora B plays an indispensible role in this process.

To uncover how hnRNPC2 repressed the expression of the Aurora B protein, we first examined Aurora B mRNA and found that it was increased in hnRNPC2-overexpressing cells. This may be due to the function of hnRNPC2 that binds with AU-rich or U-rich elements to stabilize mRNA (7,8). The results indicate that the repression of the Aurora B protein has no direct correlation with mRNA transcription and stability. We studied eIF4E, which is fundamental to the initiation of protein synthesis by binding with the 5′ terminal cap structure of mRNA (3,33,42). eIF4E is a presumptive oncogene and is frequently elevated in tumor cells with an association with a poor prognosis (43,44). In our results, Aurora B mRNA bound more hnRNPC2 and less eIF4E in hnRNPC2-overexpressing cells, suggesting that the repression of Aurora B, caused by overexpressed hnRNPC2, contributed to translational initiation inhibition. Furthermore, we found that hnRNPC2 bound more eIF4E in hnRNPC2-overexpressing SMMC-7721 cells. Thus, we postulate that hnRNPC2 inhibits eIF4E binding to Aurora B mRNA by binding with eIF4E, which may then repress eIF4E activity. Even so, there are still many details of the mechanism to be elucidated. For example, it remains unclear whether hnRNPC2 directly binds with eIF4E and whether other translational initiation factors participate in the process. These questions are worth further investigation.

Repression of Aurora B by hnRNPC2-induced multinucleation results in cell death and overexpression of hnRNPC2 accelerates the cell proliferative rate in SMMC-7721 cells. Previous reports have shown that Aurora B is increased to promote cell proliferation, while inhibition of its activity by specific inhibitors reduces the cell proliferative rate in certain tumors, which therefore was treated as a potent therapeutic target and prognostic marker (45,46). However, in certain tumors the expression of Aurora B also decreases with increased cell proliferative rate (41). However, in this study the overexpressed hnRNPC2 repressed Aurora B expression in hepatocellular carcinoma cells; however, the cell proliferative rate is still elevated, which indicates that hnRNPC2 is involved in other routes of gene regulation. In fact, hnRNPC is also treated as a potent therapeutic target and prognostic marker in tumors; its high expression in tumors indicates fast cell proliferation, high infiltration and invasion, poor therapeutic effect and high recurrence rate following surgery (2,3,11). Therefore, there is more to understand about the full function of hnRNPC2 in tumorigenesis and its progression.

In conclusion, we found that the expression of hnRNPC2 is positively correlated with multinucleation and proliferation in hepatocellular carcinoma cells. Since both are characteristics of tumors and are positively correlated with tumor grade, we propose that hnRNPC2 may be treated as a potential target for hepatocellular carcinoma cell diagnosis and treatment.
